# FFF-Printed PET and PMMA for Provisional Restorations: An In Vitro Evaluation of Mechanical Properties, Dimensional Accuracy, and Bonding Behavior

**DOI:** 10.3390/polym18091125

**Published:** 2026-05-02

**Authors:** Julia Gmeiner, John Meinen, Moritz Hoffmann, Bogna Stawarczyk

**Affiliations:** 1 Material Science Unit, Department of Prosthetic Dentistry, LMU University Hospital, LMU Munich, Goethestraße 70, 80336 Munich, Germany; john.meinen@med.uni-muenchen.de (J.M.); moritz.hoffmann@med.uni-muenchen.de (M.H.); bogna.stawarczyk@med.uni-muenchen.de (B.S.); 2 Biomaterials and Technology, Department Research, University Center for Dental Medicine Basel UZB, University of Basel, Mattenstraße 40, 4058 Basel, Switzerland

**Keywords:** 3D-printing, FFF, DLP, PMMA, PET, fracture load, dimensional accuracy

## Abstract

The purpose of this in vitro study was to evaluate the mechanical performance, dimensional accuracy, and bonding behavior of fused filament fabrication (FFF)-printed provisional restorations made from polymethyl methacrylate (PMMA) and polyethylene terephthalate (PET), and compare them with digital light processing (DLP)-printed and computer-aided numerical control (CNC)-milled ones. Occlusal veneers (OV), posterior crowns (PC), and anterior crowns (AC) (*n* = 30) were fabricated using FFF (PMMA, PET), DLP (acrylate), and CNC (PMMA) to assess initial fracture load (IFL). To determine reproducibility three restorations of each group were scanned and compared with each other; to determine printing accuracy the scanned restorations were compared with the STL generated for manufacturing. For shear bond strength (SBS) testing, 72 PMMA (FFF) specimens were conditioned with either Monobond Plus (MP) or Visiolink (VL) and bonded with acrylic cylinders using a dual-cure luting composite (Variolink Esthetic DC). Half of each group underwent thermocycling (10,000 cycles, 5 °C/55 °C, 30 s dwell time); the remainder was tested initially. Additionally, 48 FFF-printed PC were fabricated from PET and PMMA to investigate the fracture load in relation to the adhesive material (FL). PMMA crowns were conditioned with MP (*n* = 16) or VL (*n* = 16) and bonded with Variolink Esthetic DC. PET crowns were cemented with either Meron (ME) or Ketac Cem Plus (KE). Half of the PMMA and all PET crowns were subjected to masticatory simulation (1,200,000 cycles, 5 N, 5 °C/55 °C, 60 s dwell). Data were analyzed using Kolmogorov–Smirnov, Kruskal–Wallis, and Mann–Whitney U tests, including IFL, SBS and FL parametric tests, and comparisons were carried out using an independent *t*-test (α = 0.05). FFF-fabricated restorations showed the lowest fracture load values and CNC-fabricated the highest (*p* < 0.001). OV fabricated via DLP and CNC exhibited the highest fracture load (*p* < 0.001). For FFF, PC demonstrated the highest values (*p* < 0.028), whereas AC showed the lowest fracture load values (*p* < 0.001). VL showed higher initial SBS than MP (*p* < 0.001) and no impact on aging (*p* < 0.608). All MP samples showed debonding after thermocycling. Within PET and PMMA, no impact of luting/cement material on fracture load was observed (*p* = 0.116–0.282). The fracture load decreased after masticatory simulation (MP-PMMA: *p* < 0.001, VL-PMMA: *p* = 0.27). DLP-fabricated restorations showed the highest reproducibility and printing accuracy. CNC and FFF-PET showed comparable values. FFF-PMMA showed the greatest deviations. CNC-fabricated provisional restorations exhibited the highest fracture load. AC presented the lowest fracture load values. DLP provided the highest reproducibility and accuracy. VL achieved superior bonding to PMMA surfaces. Thermomechanical aging significantly reduced fracture load in both PET and PMMA restorations, regardless of luting material.

## 1. Introduction

Digital manufacturing workflows have transformed the field of dentistry by offering enhanced efficiency, precision, and versatility across multiple disciplines, including prosthodontic, oral surgery, implantology, orthodontics, endodontics, and periodontology [1,2,3,4,5,6]. Additive manufacturing (AM), commonly known as three-dimensional (3D) printing, is a layer-by-layer fabrication technique [1,2]. In dentistry, vat photopolymerization—which encompassing stereolithography (SLA) and digital light processing (DLP)—remains the most widely used AM method [3,4,7]. Vat photopolymerization, however, requires validated post-processing protocols [5,8]. AM facilitates the fabrication of geometrically complex restorations while reducing material waste and production costs compared with subtractive techniques [1,2,3,4,8,9]. Fused filament fabrication (FFF) is an extrusion-based AM technique, in which a thermoplastic filament is heated and extruded through a nozzle, depositing material layer by layer onto a build platform, predominantly used in non-industrial settings because of its simplicity and low cost [5,6,7,10]. FFF presents limitations, including reduced dimensional accuracy, inferior surface quality, and challenges in thermal gradient control, which currently limit its clinical application [5,10,11]. To date, FFF has been primarily used to produce dental models, including diagnostic models, but recent research highlights its potential in the fabrication of models of aligner production models [6,10]. However, little is known about the suitability of FFF for fabricating dental restorations, particularly when using materials such as polymethyl methacrylate (PMMA) and polyethylene terephthalate (PET).

Provisional restorations play an essential role in clinical dentistry. They protect pulpal and periodontal tissues after tooth preparation, restore occlusal function and esthetics, and facilitate phonation [12,13,14]. Indirect single-tooth restorations include crowns, partial crowns and veneers. These can be applied in both anterior and posterior regions, depending on function and esthetics. Occlusal veneers, inlays, and onlays are typically used in the posterior region. Materials used for provisional restorations must possess sufficient mechanical properties and must be biocompatible to minimize adverse tissue reactions [15]. Reported masticatory force values vary in the literature, influenced by the remaining dentition and the presence of parafunctional habits; mean occlusal forces are estimated at approximately 216 newtons (N)–382 N in the posterior region and 85 N–168 N in the anterior region [16,17,18]. However, maximum bite forces can be considerably higher and may reach approximately 600 N–850 N in the molar region [19].

PMMA has long been established for use in short- and long-term provisional restorations and denture bases due to its favorable mechanical properties and low cytotoxicity [4,20]. Prefabricated PMMA computer-aided design and computer-aided manufacturing (CAD-CAM) blocks have increasingly been used for the CNC (computer numerical control) milling of provisional restoration and denture bases. CAD-CAM blocks are produced under controlled conditions, high temperature and pressure, offering improved mechanical properties compared to conventionally processed PMMA [21,22,23]. PET is commonly used for occlusal splints, orthodontic retainers, and custom trays [24,25,26,27,28,29]. Due to its established biocompatibility and high recyclability, polyethylene terephthalate glycol (PETG) is increasingly considered a sustainable option for dental use [26,30]. In the context of fused filament fabrication, both materials exhibit thermoplastic behavior, enabling extrusion and layer-by-layer deposition with sufficient interlayer bonding [11,31]. Therefore, both materials were selected to evaluate their suitability for fused filament fabrication of provisional restorations in comparison with established manufacturing techniques. As the molecular weight may influence mechanical properties and bonding behavior, the exact molecular weight of the materials was not specified by the manufacturers. However, the literature reports typical molecular weight ranges of approximately 80,000–350,000 g/mol for PMMA and 30,000–80,000 g/mol for PET-based polymers, depending on formulation and processing conditions [32,33,34]. Various cementation strategies are available for provisional restorations, depending on the intended duration of use and material selection. These include conventional temporary cements as well as self-etching, dual-cure, and composite-based luting materials [35]. However, existing studies are often limited to individual materials or isolated properties, and comprehensive data integrating mechanical performance, dimensional accuracy, and bonding behavior across different manufacturing techniques remain scarce. While general differences between manufacturing techniques have been previously reported, the present study provides a more differentiated evaluation by directly comparing material–process combinations and integrating these parameters under thermomechanical aging conditions. This approach allows the identification of material-specific limitations and enables a clinically relevant classification of FFF-based restorations beyond general performance trends. Therefore, the aim of this study was to compare FFF-printed provisional restorations made from PMMA and compare them with DLP-printed or CNC-milled ones, focusing on the mechanical properties, dimensional accuracy, and bonding behavior. The tested null hypothesis was that neither the fabrication method nor the restoration type nor the material would significantly influence the initial fracture load of the provisional restorations. Luting would not significantly influence shear bond strength, and neither bonding, luting material, nor aging would significantly influence the fracture load of FFF-fabricated posterior crowns.

## 2. Materials and Methods

### 2.1. Manufacturing of Specimens

All specimens were fabricated using FFF, DLP and CNC (Table 1 and Table 2) and subsequently freed from support and connector structures. Before initial investigation, specimens were stored for 24 h at 37 °C in an incubator (HERAcell 150, Heraeus Kulzer GmbH, Hanau, Germany). Fracture load and shear bond strength were assessed using a universal testing machine (Zwick/Roell 1445 RetroLine, ZwickRoell GmbH & Co. KG, Ulm, Germany) operating at a crosshead speed of 1 mm/min.

### 2.2. Initial Fracture Load (IFL)

**Figure 1:**
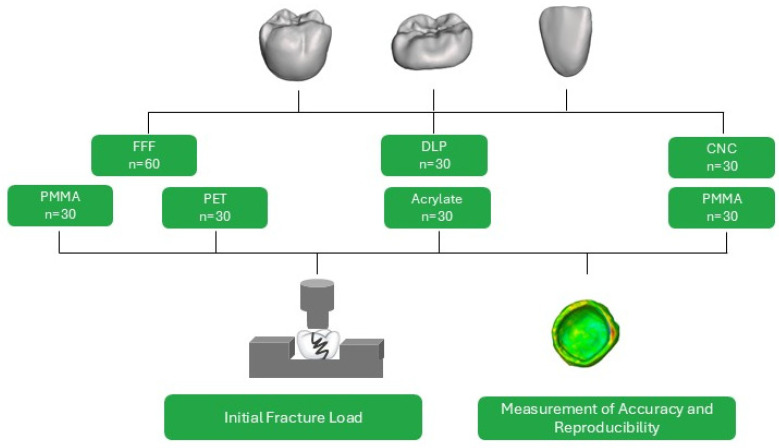
Study workflow and testing protocol. Visualization of the initial fracture load testing process for different provisional restorations (posterior crown, occlusal veneer, anterior crown) and the digital analysis method used to evaluate accuracy and reproducibility.

**Figure 2:**
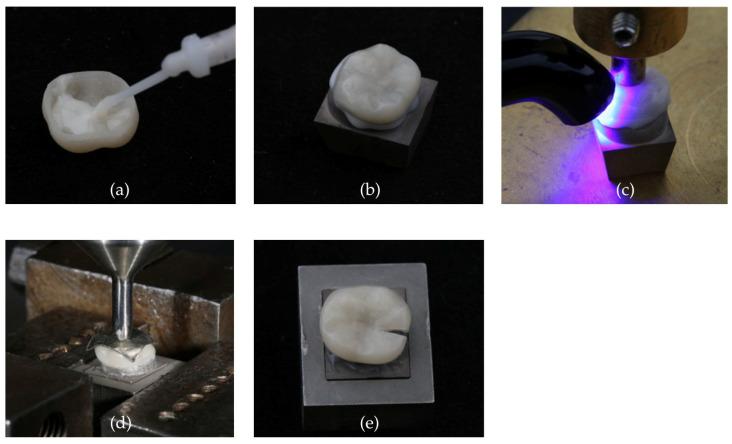
(**a**) Injecting luting material into PC; (**b**) luting PC onto CoCr abutment; (**c**) light-curing PC in fixation aid; (**d**) fracture load testing of PC; (**e**) fractured PC.

Three types of restorations were evaluated: occlusal veneers (OV), posterior crowns (PC), and anterior crowns (AC) (Figure 1). All restorations were fabricated using PET and PMMA for FFF fabrication with a build angle of 90° in mesiodistal orientation. Resin printing (DLP) acted as well as milled restorations (CNC), as the control group. Each restoration type was prepared on a typodont model (Frasaco model, frasaco GmbH, Tettnang, Germany) and scanned using a desktop scanner (CeramillMap 400, Amann Girrbach AG, Koblach, Austria), after which the restoration was digitally designed to replicate the natural tooth morphology. STL files were generated using CeramillMind (Amann Girrbach AG, Koblach, Austria). To determine reproducibility and accuracy, three restorations of each group were scanned (CeramillMap 400). The restorations were coated with Arti-Spray^®^ Occlusion Spray (Bausch GmbH & Co. KG, Cologne, Germany), positioned on a spacer platform (10 mm) and adjusted with moldable silicone. While the use of occlusal spray adds an additional surface layer that may slightly influence absolute surface measurements, the same procedure was applied to all specimens to ensure comparability. Then, the STL dataset was imported to ZEISS INSPECT Optical 3D (Carl Zeiss AG, Oberkochen, Germany) for surface comparison. To determine accuracy, the STL dataset of the scanned restoration was compared with the original STL dataset generated for manufacturing. The imported STL files were aligned with an automatic local best fit. Subsequently, a surface-based nominal–actual comparison was performed. Dimensional accuracy was evaluated based on the distribution of deviations, the maximum positive and negative deviations derived from the color scale of the deviation map. The deviation color scale was defined using a 3-sigma criterion. To determine reproducibility, pairwise comparisons of the scanned restorations were performed, analogous to the procedure described for accuracy evaluation. Prior to fracture load measurements, the restorations were polished using a polishing motor (KaVo EWL, KaVo Dental GmbH, Biberach an der Riß, Germany). Polishing consisted of two steps: pre-polish with pumice in combination with Bimssep (ERNST HINRICHS Dental GmbH, Oldenburg, Germany) at 1500 rpm, followed by final polishing using a polishing paste (Polishing paste blue P3, Polirapid Polishing Systems GmbH, Mörfelden-Walldorf, Germany) at 3000 rpm. All restorations were fixed on Cobalt-Chrome-Molybdenum (CoCrMo) alloy abutments (CeramillSintron, Amann Girrbach AG, Koblach, Austria) using dual-curing, self-adhesive luting resin composite (Bifix SE universal, Voco GmbH, Cuxhaven, Germany) with the help of a fixation aid applying a force of 10 N. Restorations were light-cured (Elipar DeepCure-S, 3M, St. Paul, MN, USA, wavelength range 430–480 nm, irradiance 1470 mW/cm^2^) for 20 s from each side (occlusal, mesial, distal, buccal, lingual). To determine the initial fracture load (IFL) a 6 mm diameter steel stamp with an intermediate tinfoil (0.3 mm thick) (DENTAURUM GmbH & Co. KG, Ispringen, Germany) was used. For OV and PC the stamp was aligned in the occlusal center to achieve multipoint contact, whereas for AC, the stamp was positioned centrally on the incisal edge (Figure 2).

### 2.3. Shear Bond Strength (SBS)

**Figure 3:**
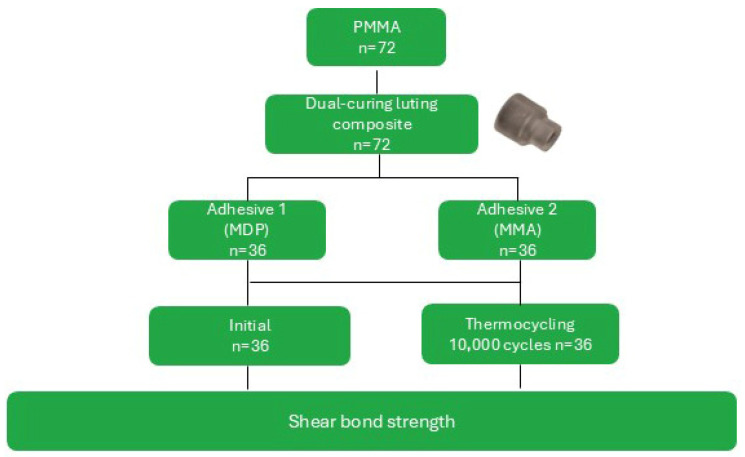
Study design of the shear bond strength testing. Overview of specimen preparation using FFF-printed PMMA substrates, surface conditioning with two different adhesive systems, aging protocols, and mechanical SBS testing protocol. The Symbol represents the acrylic cylinder.

To determine the shear bond strength, 72 rectangular specimens (10 × 10 × 2 mm) using FFF with PMMA were printed with a 90° orientation to the building platform. The specimens were embedded (Scandiquick, SCAN-DIA GmbH, Hagen, Germany), ground flat and air-abraded with 50 µm Al_2_O_3_ at 0.1 MPa for 10 s at a 45° angle and a distance of 10 mm (Basic quattro IS, Renfert GmbH, Hilzingen, Germany), followed by cleaning in an ultrasonic bath (SONOREX DIGITEC DT 31H, BANDELIN electronic GmbH & Co. KG, Berlin, Germany) with distilled water for 3 min. The specimens were divided into two surface condition groups. Group 1 (MP; *n* = 36) was conditioned with a universal primer (Monobond Plus, Ivoclar Vivadent AG, Schaan, Liechtenstein). The primer was applied for 60 s and then air-dried. Group 2 (VL; *n* = 36) was conditioned with an adhesive (Visiolink, bredent GmbH & Co KG, Senden, Germany) and light-cured for 3 min (bre.Lux PowerUnit 2, bredent GmbH & Co. KG, Senden, Germany, wavelength range 370–500 nm; irradiance: 1000 mW/cm^2^–1700 mW/cm^2^). Then, acrylic cylinders (N = 72) (SD Mechatronik GmbH, Feldkirchen-Westerham, Germany) were luted to the substrate using dual-curing luting composite (Variolink Esthetic DC, Ivoclar Vivadent AG, Schaan, Liechtenstein), and light-cured for 20 s from the top and four circumferential directions (Elipar DeepCure-S). Half of each group underwent thermocycling (10,000 cycles, 5 °C/55 °C, 30 s dwell time, SD Mechatronik GmbH, Feldkirchen-Westerham, Germany), and the other half of each group was measured initially. Shear bond strength was measured according to ISO 29022 using a universal testing machine equipped with a shear test device at a crosshead speed of 1 mm/min (Figure 3). After SBS testing, the specimens were analyzed using a digital light microscope (VHX-970 F, Keyence Corp., Osaka, Japan). Fractographic evaluation was performed to determine the failure mode. Failures were classified as adhesive, cohesive, or mixed failure types.

### 2.4. Fracture Load Depending on Adhesive Material (FL)

**Figure 4:**
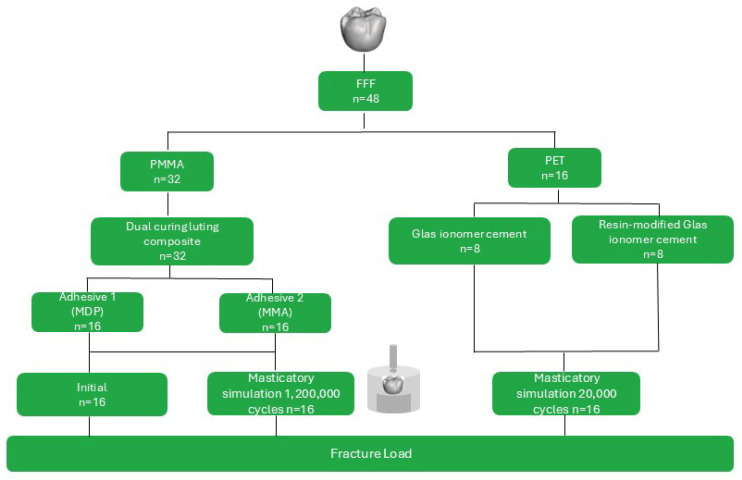
Study design of the fracture load depending on adhesive material. Overview of specimen preparation (posterior crown) using FFF-printed PMMA and PET substrates, different luting materials, surface conditioning with two different adhesive systems for PMMA substrates, aging protocols (masticatory simulation; crown in chewing simulator), and mechanical fracture load testing protocol.

To determine the fracture load depending on the adhesive material (FL), 48 posterior crowns (PC) were printed (FFF) using PET and PMMA. All PC were printed with the lingual surface facing the building platform and air-abraded with 125 µm Al_2_O_3_ at 0.1 MPa for 10 s at a 45° angle and a distance of 10 mm (Basic quattro IS). The specimens were cleaned in an ultrasonic bath with distilled water for 3 min. The PMMA PC were divided into two surface condition groups: i. MP-PMMA (*n* = 16) and ii. VL-PMMA (*n* = 16). MP-PMMA specimens were conditioned with Monobond Plus (MP), VL-PMMA with Visiolink (VL). The restorations (*n* = 32) were luted onto the polymer-based abutment (TRINIA; Bicon Europe Ltd., Madrid, Spain) using Variolink Esthetic DC analogous to the procedure described for SBS. The PC made of PET were cemented using two provisional luting materials: i. a glass ionomer cement (ME, Meron, Voco GmbH, Cuxhaven, Germany) and ii. a resin-modified glass ionomer cement (KE, Ketac Cem Plus, Solventum Germany GmbH, Neuss, Germany). Half of the PMMA specimens and all PET specimens underwent masticatory simulation (1,200,000 cycles, 5 N, 5 °C/55 °C, 60 s dwell time, Chewing Simulator CS-4.10; SD Mechatronik GmbH, Feldkirchen-Westerham, Germany), and the other half of the PMMA specimens were measured initially. To determine the FL a 6 mm diameter steel stamp with intermediate tinfoil (0.3 mm thick) was used. The stamp was aligned in the occlusal center to achieve multipoint contact (Figure 4). After FL testing, the failure modes were visually assessed and classified as fracture, debonding, or combined failure based on the integrity of the restoration and its retention on the abutment.

### 2.5. Statistical Analysis

Statistical analysis was performed using IBM SPSS Statistics 26.0 (IBM Corp., Armonk, NY, USA). Descriptive statistics (mean ± standard deviation and 95% confidence intervals) were calculated for all groups.

Data distribution was assessed using the Kolmogorov–Smirnov test. Based on the distribution, either parametric or non-parametric tests were applied.

For non-normally distributed data (initial fracture load), group comparisons were performed using the Kruskal–Wallis test followed by pairwise comparisons with the Mann–Whitney U test.

For normally distributed data (shear bond strength and fracture load depending on luting material and aging), comparisons between groups were performed using independent *t*-tests.

The level of statistical significance was set at α = 0.05. Due to the exploratory nature of this study, no adjustment for multiple testing was applied.

## 3. Results

### 3.1. Results of Initial Fracture Load (IFL)

**Figure 5:**
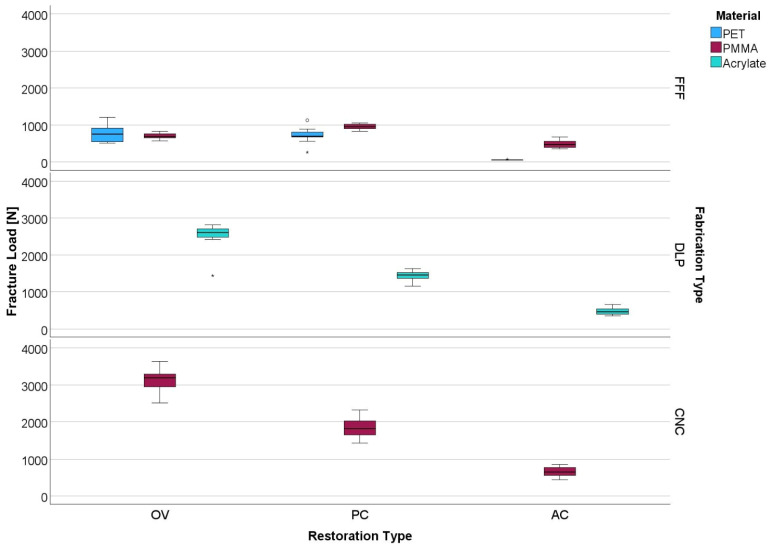
Boxplots showing fracture load of different restoration types, fabrication types and materials. ○ represents outliers and * represents extreme outliers.

FFF-fabricated OV, and PC and AC showed the lowest and CNC-fabricated restorations the highest fracture load values (*p* < 0.001). Among the evaluated fabrication techniques, OV fabricated with DLP and CNC exhibited the highest fracture load (*p* < 0.001) for FFF, and PC demonstrated the highest values (*p* < 0.028). AC showed the lowest fracture load values (*p* < 0.001). CNC-PMMA showed higher values for all restoration types than FFF-PMMA (*p* < 0.001). Within the FFF-fabricated restorations, PMMA showed higher values for PC and AC (*p* < 0.001) (Table 3, Figure 5).

### 3.2. Results of Shear Bond Strength (SBS)

**Figure 6:**
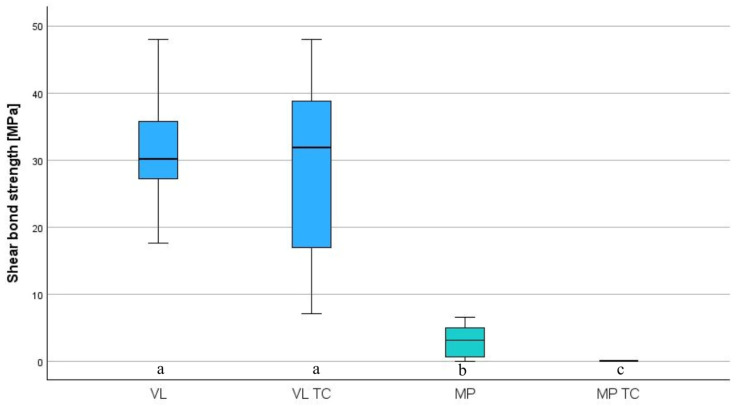
Boxplots showing shear bond strength of different surface conditioning initially and after thermocycling (TC) (*n* = 18 per group). a, b, c indicate significant differences between the groups.

**Figure 7:**
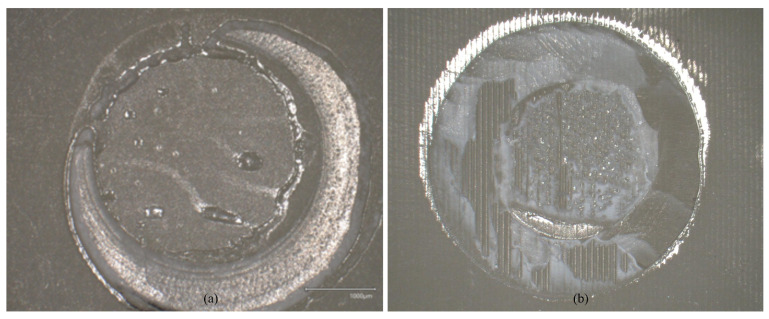
Mixed failure modes showing shear bond strength after thermocycling (Keyence VHX970-F, Keyence Corp., Osaka, Japan): (**a**) Monobond Plus (initial); (**b**) Visiolink (initial).

VL showed higher results than MP (*p* < 0.001) regarding the initial SBS. VL showed no influence on aging (*p* < 0.608). MP showed debondings of all specimens during thermocycling (*p* < 0.001) (Table 4, Figure 6 and Figure 7). The distribution of failure modes is summarized in Table 5.

### 3.3. Results of Fracture Load Depending on Adhesive Material (FL)

**Figure 8:**
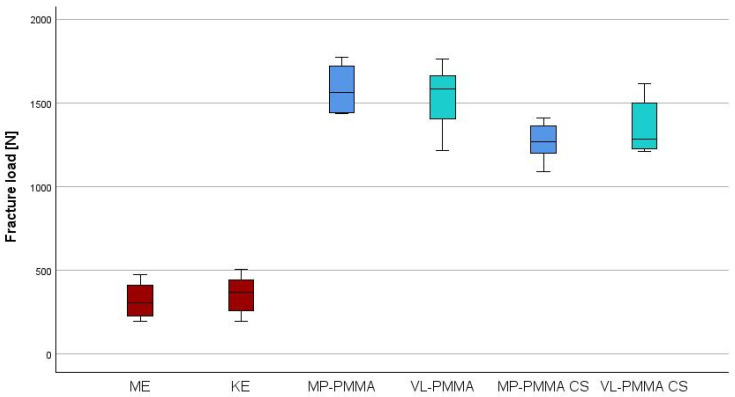
Boxplots showing fracture load depending on luting material initially and after chewing simulation (CS).

**Figure 9:**
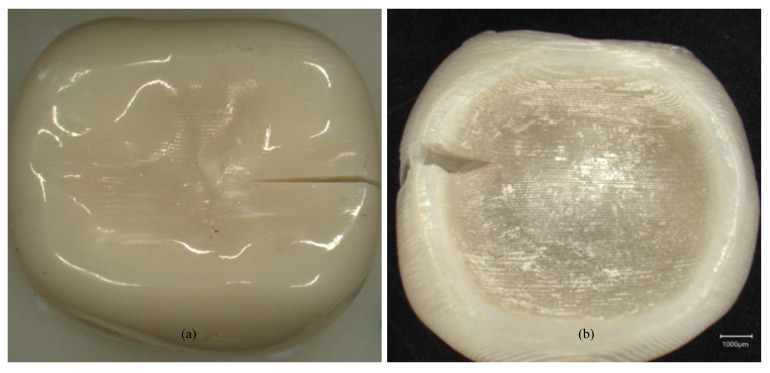
Failure modes after fracture load testing (Keyence VHX970-F): (**a**) fractured MP-PMMA crown; (**b**) combined failure ME-PET crown.

The FL of PC made of PET showed comparable values for ME and KE (*p* = 0.265). Within the PC made of PMMA, no difference regarding the fixation material was observed in relation to the FL initially (*p* = 0.282) and after the chewing simulation (*p* = 0.116). For both luting materials, stability decreased after masticatory simulation (MP-PMMA: *p* < 0.001, VL-PMMA: *p* = 0.27) (Table 6, Figure 8). PMMA and PET KE specimens exhibited predominantly fracture-dominated failures before and after thermocycling, whereas PET ME showed a higher proportion of combined failures (Table 7, Figure 9).

### 3.4. Results of Reproducibility and Accuracy

**Figure 10:**
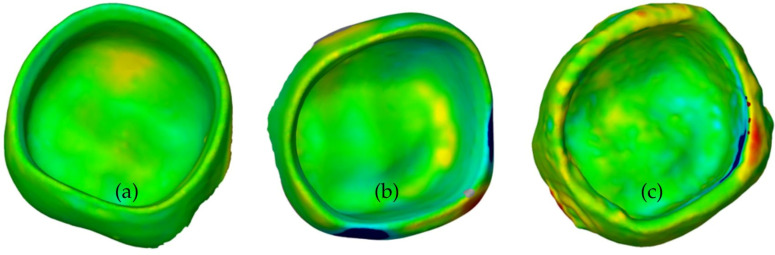
(**a**) Measurement of reproducibility of DLP-printed PC; (**b**) measurement of reproducibility of CNC-printed PC; (**c**) measurement of reproducibility of FFF-printed PC. Color representation of deviations: green indicates no deviation, blue indicates negative deviation, and yellow to red indicate positive deviation, and grey indicates missing data.

DLP-fabricated restorations showed the highest reproducibility and printing accuracy. CNC and FFF-PET showed comparable values. FFF-PMMA showed the greatest deviations. DLP showed most deviations on the basal surface. CNC showed the most deviations on the outer surface. The deviations of the FFF-fabricated restorations were irregular (Figure 10).

## 4. Discussion

The aim of this study was to evaluate the mechanical properties, dimensional accuracy, and bonding behavior of provisional restorations fabricated using FFF, in comparison with DLP-printed and CNC manufacturing techniques. The null hypotheses were rejected, as fabrication method, restoration type, and material significantly influenced the investigated parameters. Although general trends observed in this study, such as the superior mechanical performance of CNC-milled restorations and the higher accuracy of DLP compared to FFF, are consistent with the existing literature [6,9,36,37], the present investigation provides a comprehensive evaluation by integrating mechanical performance, dimensional accuracy, and bonding behavior within a single experimental framework. In particular, the findings highlight material- and process-specific limitations of FFF-manufactured restorations, especially with regard to dimensional accuracy, bonding performance, and stability after thermomechanical aging, which are critical for clinical application.

CNC-fabricated restorations exhibited the highest fracture load across all restoration types, which can be attributed to the industrial production conditions of prefabricated PMMA discs, resulting in high dense and homogeneous material structures with enhanced mechanical properties [21,22,23]. In contrast, FFF-fabricated restorations showed the lowest fracture load values. This may be attributed to the layer-by-layer deposition process inherent to FFF, which leads to anisotropic mechanical behavior and potentially weaker interlayer bonding. These interfacial regions may act as preferential sites for crack initiation and propagation under load [38]. The non-rectangular geometry of extruded beads promotes the formation of interstitial voids between adjacent filaments, which in turn plays a key role in the anisotropic mechanical response of FFF structures [39]. In FFF, the rapid cooling of deposited filaments restricts the time window for polymer chain mobility and interdiffusion. Consequently, the interface temperature may fall below the glass transition temperature before sufficient molecular entanglement and neck growth can be established, resulting in reduced interlayer bonding [40]. These interpretations should be considered as plausible mechanisms, as no direct microstructural analyses were performed.

In addition, several process-related factors influence the mechanical performance of FFF restorations, including print orientation, layer height, infill density and type, nozzle diameter, and extrusion and building platform temperature [1,5,11,38]. The observed variability in fracture resistance and dimensional accuracy may therefore be attributed to these process-dependent characteristics.

Since provisional restorations are required to withstand functional masticatory forces during clinical use, fracture resistance represents a critical parameter for their clinical applicability [23,41]. Maximal bite forces are estimated to range from approximately 85 to 168 N in the anterior region and up to 600–850 N in the posterior region [16,18,19]. Based on these reference values, the fracture load results of the present study allow a clinically oriented classification of the investigated materials. Restorations exhibiting fracture load values exceeding posterior bite force ranges (>600 N) may be considered suitable for broader provisional indications, including posterior regions. Accordingly, FFF-PMMA restorations exceeded physiological bite forces and may therefore be considered mechanically sufficient for a wide range of provisional applications, particularly when adhesively luted. In contrast, FFF restorations—especially PET-based ones—with lower fracture load values appear more suitable for anterior regions or short-term and low-load provisional applications. These thresholds provide a practical framework for selecting suitable materials depending on the expected functional load.

Among all restoration types, anterior crowns demonstrated the lowest fracture resistance, which is primarily attributed to their reduced wall thickness and unfavorable loading geometry. Axial loading on the incisal edge induces tensile stresses on the palatal surface, promoting earlier crack initiation. However, this may be of limited clinical relevance due to lower functional loads in the anterior region [16].

The comparatively lower mechanical performance of PET can be explained by its material characteristics. PET exhibits more pronounced plastic deformation behavior under load, which may limit its fracture resistance. The literature reports lower elastic modulus (Duran: 1.35 GPa–1.70 GPa) and strength values (Duran: 49.00 MPa–53.50 MPa) for PET-based materials compared to PMMA (strength: 80 and 110 MPa), supporting the findings of the present study [20,26,28].

Regarding dimensional accuracy, DLP-fabricated restorations demonstrated the highest precision, while CNC and FFF-PET showed comparable results. FFF-PMMA restorations exhibited the largest and irregular deviations, which may be attributed to material-specific behavior during extrusion and cooling, as well as to the layer-based fabrication process. It should be emphasized that differences in dimensional accuracy may introduce geometric variations, potentially leading to stress concentration and influencing mechanical performance.

However, these deviations reflect inherent characteristics of the respective manufacturing techniques and should therefore be considered as part of the investigated material/process combinations rather than as experimental bias. Since all specimens were fabricated and tested under standardized conditions, a consistent comparison between groups was ensured. Nevertheless, the potential influence of these deviations should be considered when interpreting the results.

Regarding the bonding behavior, the adhesive system Visiolink (VL), containing methyl methacrylate (MMA), showed significantly higher SBS values compared to Monobond Plus (MP). This can be attributed to the chemical interaction between MMA and the PMMA substrate, enabling bonding to the polymer network formation and improved adhesion. In contrast, MP primarily relies on functional monomers such as MDP, which are more effective for bonding to oxide ceramics and metals [22]. This is supported by the failure mode analysis. MP predominantly exhibited mixed failures before and after thermocycling. All MP TC specimens debonded during thermocycling, indicating insufficient interfacial stability under aging conditions. In contrast, VL remained bonded after thermocycling and showed a more heterogeneous failure pattern, including adhesive, mixed, and cohesive failures. These findings are consistent with the SBS values observed for VL.

The relatively high variability observed in certain groups, particularly in the VL thermocycled group, may be attributed to multiple factors, including material heterogeneity, surface treatment variability, and degradation process at the bonding interface during thermomechanical aging. In addition, anisotropic structure of FFF-fabricated materials may contribute to increased variability in bonding performance. Despite this dispersion, consistent trends between groups were observed, supporting the overall validity of the findings. Conventional glass ionomer cements such as Meron (ME) rely solely on an acid–base reaction, whereas resin-modified glass ionomer cements like KE incorporate hydrophilic methacrylate monomers that introduce a secondary light-activated polymerization [35]. PET exhibits smooth, hydrophobic surfaces with low surface free energy, resulting in limited wettability and minimal chemical interaction with dental luting agents [29]. This explains why conventional and resin-modified GICs showed no measurable difference in FL for the PET PC.

Thermomechanical aging resulted in a reduction in fracture load for both PET and PMMA restorations. For PMMA, this can be explained by water sorption and plasticization effects, which reduce intermolecular interactions and facilitate subcritical crack growth under cycling stress [23]. For PET, increased plastic deformation and potential hydrolytic degradation may contribute to reduced mechanical stability. These findings are consistent with the observed failure modes, where PET restorations exhibited a higher proportion of combined failures, partly associated with plastic deformation of the material, indicating a relevant contribution of the adhesive interface to the overall failure behavior. In contrast, PMMA restorations showed predominantly fracture-dominated failures, suggesting that failure was mainly governed by the mechanical properties of the material rather than the adhesive interface.

Several limitations of this study should be considered. Differences in dimensional accuracy may have influenced mechanical performance by introducing geometric variations. In addition, relatively small subgroups sizes may affect statistical power and generalizability. Given the exploratory design and the number of statistical comparisons performed, no formal correction for multiple testing was applied. Therefore, the results should be interpreted with caution, with emphasis placed on consistent trends rather than isolated statistically significant findings.

Furthermore, the use of scan spray during surface digitization may introduce minor measurement bias, although it was applied consistently across all specimens to ensure comparability. Additionally, no scanning electron microscopy (SEM) analysis was performed to further characterize surface morphology, bonding interfaces, or failure mechanisms. Such analyses could provide deeper insight into the underlying mechanisms and should be addressed in future studies. Similarly, Micro-CT analysis was not performed and could provide additional information on internal structural deviations.

From a clinical perspective, the results indicate that FFF-manufactured restorations may represent a viable option for provisional applications, particularly when material selection and processing parameters are carefully considered. However, limitations in dimensional accuracy and bonding performance after thermomechanical aging may restrict their use to selected indications with lower mechanical and precision requirements. These findings move beyond confirmation of expected trends by defining the clinical applicability and limitations of FFF-based restorations in a more nuanced manner.

## 5. Conclusions

Within the limitations of this in vitro study, the following conclusions can be drawn. CNC-fabricated provisional restorations exhibited the highest fracture load, while FFF-printed restorations demonstrated the lowest fracture load. Anterior crowns presented the lowest fracture load values, though this may be clinically acceptable due to lower occlusal loading in the anterior region. DLP-printed restorations provided the highest reproducibility and accuracy; FFF-PET-printed restorations showed dimensional consistency similar to CNC, while FFF-PMMA-printed restorations displayed the greatest deviations. VL achieved superior bonding to FFF-PMMA surfaces compared to MP and maintained bond strength after thermocycling. Thermomechanical aging significantly reduced fracture load in both PET and PMMA restorations, regardless of the luting material.

While FFF remains in the early stages of clinical adoption, this study highlights its potential for provisional applications, provided that material selection, surface treatment, and aging behavior are taken into account. The proposed fracture load thresholds may support clinical decision-making when selecting provisional materials based on expected functional load.

## Figures and Tables

**Table 1:** Overview of fabrication workflows and materials used.

Fabrication Method	Manufacturing Device	Software	Post-Processing Protocol	Material	Lot Number
FFF	SIMPLEX 2 SX (Renfert GmbH, Hilzingen, Germany)	PrusaSlicer (Prusa Research a.s., Prague, Czech Republic)		PMMA (The.r.mo.bridge, Pressing Dental S.r.l., City of San Marino, San Marino)	00560
				PET (ELDY FILAMENT, DentalPlus GmbH, Bensheim, Germany)	2405032
DLP	ASIGA UV MAX (Asiga Pty Ltd., Sydney, Australia)	Asiga composer software (Asiga Pty Ltd., Sydney, Australia) Layer thickness 50 µm	Washing: 3 and 2 min ultrasonic bath (SONOREX DIGITEC DT 31H, BANDELIN electronic GmbH & Co KG, Berlin, Germany) with 99% isopropanol Post-polymerization: 2 × 1500 flashes under nitrogen atmosphere (Otoflash G171, NK Optik GmbH, Baierbrunn, Germany)	VarseoSmile CrownPlus resin (Bego GmbH & Co. KG, Bremen, Germany)	601374
CNC	CeramillMotion 2 (Amann Girrbach AG, Koblach, Austria)	CeramillMind (Amann Girrbach AG, Koblach, Austria)		Telio CAD LT (Ivoclar Vivadent AG, Schaan, Liechtenstein)	YBDS9F

**Table 2:** Printing parameters for FFF-fabricated PET and PMMA specimens, including filament characteristics, processing temperatures, printer settings, and software used.

Filament	PMMA	PET
Diameter	1.75 mm	1.75 mm
Printing speed	30–130 mm/s	40–130 mm/s
Nozzle diameter	0.25 mm	0.25 mm
Z-resolution	0.1 mm	0.1 mm
Heated building platform	110 °C	85 °C
Extrusion temperature	265 °C	280 °C
Slicing software	PrusaSlicer-2.8.0	
Printer	SIMPLEX 2 SX	

**Table 3:** Descriptive statistics of initial fracture load of three different restoration types [mean ± standard deviation (SD) and 95% confidence interval (CI)].

Restoration Type	Fabrication Type	Material	Mean ± SD [N]	CI (95% CI) [N]
Occlusal Veneer	FFF	PET	778 ± 219	(621, 935)
		PMMA	691 ± 81	(633, 750)
	DLP	Acrylate-based Resin	2512 ± 395 *	(2230, 2795)
	CNC	PMMA	3142 ± 308	(2922, 3363)
Posterior Crown	FFF	PET	718 ± 222	(559, 877)
		PMMA	952 ± 77	(896, 1007)
	DLP	Acrylate-based Resin	1427 ± 163	(1310, 1544)
	CNC	PMMA	1841 ± 255	(1659, 2023)
Anterior Crown	FFF	PET	52 ± 6 *	(47, 57)
		PMMA	482 ± 104	(407, 557)
	DLP	Acrylate-based Resin	486 ± 104	(412, 560)
	CNC	PMMA	661 ± 140	(561, 761)

* Deviation from normal distribution.

**Table 4:** Descriptive statistics of shear bond strength [mean ± standard deviation (SD) and 95% confidence interval (CI)].

Fabrication Type	Material	Bonding	Initial/After TC	Mean ± SD [N]	CI (95% CI) [N]
FFF	PMMA	Visiolink	initial	31 ± 7	(27, 34)
			after TC	29 ± 12	(23, 35)
		Monobond Plus	initial	3 ± 2	(2, 4)
			after TC	0 ± 0	(0, 0)

**Table 5:** Distribution of failure modes based on fractographic analysis.

Fabrication Type	Material	Bonding	Initial/After TC	Adhesive *n* (%)	Cohesive *n* (%)	Mixed *n* (%)
FFF	PMMA	Visiolink	initial	3 (16.67%)	3 (16.67%)	12 (66.67%)
			after TC	3 (16.67%)	6 (33.33%)	9 (50.00%)
		Monobond Plus	initial	2 (11.11%)	0	16 (88.89%)
			after TC	2 (11.11%)	0	16 (88.89%)

**Table 6:** Descriptive statistics of fracture load depending on luting material [mean ± standard deviation (SD) and 95% confidence interval (CI)].

Fabrication Type	Material	Luting	Bonding	Initial/After CS	Mean ± SD [N]	CI (95% CI) [N]
FFF	PET	Meron	-	after CS	319 ± 106	(230, 407)
		Ketac Cem Plus	-	after CS	354 ± 113	(260, 448)
	PMMA	Variolink DC	Monobond Plus	initial	1582 ± 141	(1464, 1677)
			Visiolink	initial	1533 ± 183	(1381, 1686)
			Monobond Plus	after CS	1269 ± 112	(1175, 1362)
			Visiolink	after CS	1354 ± 158	(1222, 1486)

**Table 7:** Distribution of failure mode after fracture load testing.

Fabrication Type	Material	Bonding	Initial/After TC	Fractured *n* (%)	Debonding *n* (%)	Combined *n* (%)
FFF	PMMA	Visiolink	initial	8 (100%)	0	0
			after TC	8 (100%)	0	0
		Monobond Plus	initial	8 (100%)	0	0
			after TC	8 (100%)	0	0
	PET	Meron	initial	1 (12.5%)	1 (12.5%)	6 (75%)
		Ketac Cem Plus		5 (62.5%)	0	3 (37.5%)

## Data Availability

The data presented in this study are available on request from the corresponding author.

## Abbreviations

The following abbreviations are used in this manuscript:

FFF	Fused filament fabrication
PMMA	Polymethyl methacrylate
PET	Polyethylene terephthalate
DLP	Digital light processing
CNC	Computer-aided numerical control
OV	Occlusal veneer
PC	Posterior crown
AC	Anterior crown
IFL	Initial fracture load
SBS	Shear bond strength
FL	Fracture load depending on adhesive material
MP	Monobond Plus
VL	Visiolink
ME	Meron
KE	Ketac Cem Plus
AM	Additive manufacturing
SLA	Stereolithography
CoCroMo	Cobalt-Chrome-Molybdenum
MMA	Methyl methacrylate
